# Comparative Proteomic Analysis of Aqueous Humor, Anterior Lens Capsules, and Crystalline Lenses in Different Human Cataract Subtypes Versus Healthy Controls

**DOI:** 10.3390/proteomes13040062

**Published:** 2025-11-21

**Authors:** Christina Karakosta, Martina Samiotaki, Anastasios Bisoukis, Konstantinos I. Bougioukas, George Panayotou, Nantieznta Kyriakidou, Konstantinos Moschou, Marilita M. Moschos

**Affiliations:** 1 School of Medicine, National and Kapodistrian University of Athens, 12462 Athens, Greece; 2 Biomedical Sciences Research Center “Alexander Fleming”, 16672 Vari, Greece; 3 Bristol Eye Hospital, University Hospitals Bristol NHS Foundation Trust, Bristol BS1 2LX, UK; 4 Department of Hygiene, Social-Preventive Medicine and Medical Statistics, School of Medicine, Faculty of Health Sciences, Aristotle University of Thessaloniki, 54124 Thessaloniki, Greece; 5 Day Unit Clinic DIATHLASIS, 54627 Thessaloniki, Greece

**Keywords:** aqueous humor, anterior capsule, cataract, crystalline, lens, age, diabetes, vitrectomy, proteomics

## Abstract

Background: The aim of this study is to investigate the pathophysiology of cataract by analyzing signaling pathways in three sample types obtained from four different lens groups: age-related (ARC), diabetic (DC), post-vitrectomy cataract (PVC) and clear control lenses. Methods: Three sample types—the aqueous humor, the anterior capsule and the phaco cassette content—were collected during cataract surgery from 39 participants (ARC = 12, DC = 11, PVC = 7 and control = 9). The samples were prepared based on Sp3 protocol. The recognition and quantification of proteins were performed with liquid chromatography online with tandem mass spectrometry using the DIA-NN software. Perseus software (v1.6.15.0) was used for statistical analysis. Data are available via ProteomeXchange with identifiers PXD045547, PXD045554, PXD045557, and PXD069667. Results: In total, 1986 proteins were identified in the aqueous humor, 2804 in the anterior capsule, and 3337 in the phaco cassette samples. Proteins involved in actin and microtubule cytoskeleton organization, including ACTN4, were downregulated in all three cataract groups compared to controls. Proteins involved in glycolipid metabolic process, including GAL3ST1, GAL3ST4, and GLA, were upregulated in ARC compared to controls. Proteins involved in the non-canonical Wnt receptor signaling pathway, including FRZB, SFRP1, SFRP2, SFRP5, WNT5A, and WNT7A, were upregulated in ARC compared to DC, PVC, and controls. Conclusions: Comprehensive proteomic profiles were generated using DIA proteomics by comparing ARC, DC, and PVC versus controls. This is the first study to use phaco cassette contents to investigate cataract formation in comparison to controls. Our findings significantly enhance the current understanding of human cataract pathophysiology and provide novel insights into the mechanisms underlying cataract formation.

## 1. Introduction

Cataract remains the leading cause of visual impairment and blindness worldwide, particularly affecting the aging population [1]. Characterized by the opacification of the normally transparent crystalline lens, cataract can manifest in various morphological subtypes, including nuclear, cortical, posterior subcapsular, and mixed forms [2]. While the clinical and morphological classification of cataracts is well-established, the molecular mechanisms underlying their development and progression remain incompletely understood.

The eye offers a unique anatomical compartmentalization that facilitates targeted biochemical investigations. The aqueous humor serves as a medium for the transport of nutrients and waste, and reflects dynamic changes in the intraocular environment mirroring systemic conditions [3,4]. The anterior lens capsule, the basement membrane of the lens epithelium, acts as both a protective barrier and a modulator of cellular activity [5,6]. The crystalline lens itself, composed of highly ordered fiber cells and proteins, which do not turnover, is particularly vulnerable to oxidative stress, post-translational modifications, and proteostatic imbalance—all of which have been implicated in cataractogenesis [7].

The pathogenesis of cataract is multifactorial and varies by subtype. Age-related cataract (ARC) is largely attributed to oxidative damage and protein aggregation that accumulates over time [8]. In diabetic cataract (DC), chronic hyperglycemia contributes to metabolic stress and osmotic imbalance within lens fibers [9]. Post-vitrectomy cataract (PVC), on the other hand, is believed to result from altered intraocular oxygen gradients and exposure of the lens to oxidative stress following vitreous removal [10,11]. These subtypes likely involve overlapping, but distinct molecular pathways, yet few studies have examined their proteomic profiles in a comparative fashion.

Proteomics—the large-scale study of proteins and their functions—has emerged as a powerful approach to characterizing disease-specific molecular changes [12]. Mass spectrometry, particularly data-independent acquisition (DIA) methods, allows for sensitive, high-throughput analysis of protein expression in complex biological samples [13]. Previous proteomic investigations have revealed important insights into age-related changes in the human lens, lens epithelial cell metabolism, and stress response mechanisms [14,15]. A recent study showed that in mice with point mutations in α-, β- or γ-crystallins, the mutant proteins were unstable and reduced in level but did not accumulate in the water-insoluble fraction. Meanwhile, many of the non-mutant crystallins (including α-crystallins) did precipitate [16]. The authors concluded that the root cause of cataract here appears to be a proteome imbalance, altered proportions and interactions of crystallin proteins, rather than simply the aggregation propensity of the mutated proteins [16]. Interestingly, a another recently published study demonstrated that with increasing age, the lens proteome exhibits less intrinsic protein disorder [17]. Nonetheless, most prior work has focused on isolated tissue types or a single cataract form, limiting our understanding of how systemic conditions, intraocular surgical interventions, or aging differentially influence the lens environment.

Given the distinct pathophysiological mechanisms presumed to underlie different cataract subtypes, we hypothesized that the proteomic signatures of the aqueous humor, anterior capsule, and lens content would differ between ARC, DC, and PVC cases, and also from normal controls. By applying a data-independent acquisition (DIA) mass spectrometry strategy, our goal was to systematically characterize and compare the proteomic profiles of these three tissue types across clinical groups. Identification of subtype-specific proteomic alterations could enhance our understanding of cataract pathophysiology, reveal potential molecular biomarkers, and provide insight into shared and divergent pathways of lens degeneration. Our findings significantly enhance the current understanding of human cataract pathophysiology and provide novel insights into the mechanisms underlying cataract formation. The current study is an extension of our previous project [18].

## 2. Materials and Methods

### 2.1. Human Subjects

This study adhered to the ethical standards outlined in the Declaration of Helsinki and followed the principles of good clinical practice in research involving human participants. Written informed consent was obtained from all individuals prior to their inclusion in the study. To ensure confidentiality, all clinical data were anonymized before analysis. Ethical approval for the study was granted by the Institutional Review Board of the Hospital (Approval No. 18534/20-07-22).

Participants were recruited from the Ophthalmology Department among patients scheduled for cataract surgery. Patients were stratified into four distinct groups based on their clinical profiles. ARC group (Age-Related Cataract) included individuals over the age of 75 without a history of diabetes; DC group (Diabetic cataract) included individuals under the age of 65 with a known diagnosis of type 1 or type 2 diabetes mellitus; PVC group (Post-Vitrectomy cataract) included individuals of any age, without diabetes, who had undergone vitrectomy with gas tamponade for retinal detachment within the previous 12 months and who had clear lens prior to vitrectomy; and the control group included individuals under the age of 60, without diabetes, who underwent clear lens extraction for refractive reasons.

Eligibility criteria for ARC, DC and PVC groups included a clinical diagnosis of cataract confirmed by slit-lamp examination and best-corrected visual acuity of 20/40 or worse, while for the control group, the clarity of the lens was confirmed by two independent clinicians. Exclusion criteria for all groups included a history of ocular trauma or chronic use of corticosteroids (either systemic or topical). An additional exclusion criterion for the control group included myopia, in order to avoid selection bias, since myopia is associated with an increased risk of nuclear and posterior subcapsular cataract [19].

For each participant, a detailed medical and lifestyle history was obtained. Data collected included iris color, cataract subtype, alcohol and smoking habits, duration of sun exposure and use of sunglasses, systemic conditions (e.g., hypertension, thyroid disorders, glaucoma, age-related macular degeneration), dietary supplement use, and prior estrogen therapy.

### 2.2. Sample Collection

Three sample types—aqueous humor, anterior lens capsule, and the content of the phaco cassette—were obtained from each participant during routine cataract surgery. To ensure consistency and minimize variability in surgical handling and operative duration, all procedures were performed by the same experienced ophthalmic surgeon.

Following a standard clear corneal incision using an I-Knife keratome, approximately 0.2 mL of aqueous humor was aspirated from the anterior chamber. Once continuous curvilinear capsulorhexis was completed, the anterior lens capsule was collected.

At the end of the procedure, the content of the phacoemulsification cassette (Centurion^®^ Vision System, Alcon, Geneva, Switzerland) was collected, and this contained Balanced Salt Solution (BSS) mixed with phacoemulsified lens fragments and re-secreted aqueous humor. All collected samples were immediately stored at −80 °C (Haier Biomedical, Qingdao, China).

### 2.3. Sample Preparation

The procedures for sample preparation and data analysis were aligned with those employed in our previous project [18].

Anterior lens capsule samples were first lysed and homogenized in a buffer containing 4% sodium dodecyl sulfate (SDS) and 0.1 M dithiothreitol (DTT) in 0.1 M triethylammonium bicarbonate (TEAB). The lysates underwent two cycles of heating and sonication. From this stage onward, the sample preparation protocol was standardized across all three tissue types.

For the phaco cassette contents, 100% ethanol was added to the sample in a 1:1 volume ratio, along with 40 µL of a bead mixture per 250 µL of sample. The beads consisted of a 1:1 combination of hydrophilic and hydrophobic SeraMag carboxylate-modified magnetic particles (Cytiva, Marlborough, MA, USA). The samples were then centrifuged twice at 2200 rpm for 10 min to pellet the beads.

Protein extracts from all three sample types underwent enzymatic digestion following the Single-Pot Solid-Phase-enhanced Sample Preparation (SP3) protocol. Then, the protein-bead complexes were digested overnight at 37 °C using 0.5 µg of Trypsin/LysC mix (MS grade, Promega, Madison, WI, USA) in 25 mM ammonium bicarbonate.

The following day, peptides were purified using a modified SP3 clean-up protocol, resuspended in mobile phase A (0.1% formic acid in water), sonicated, and quantified by absorbance at 280 nm using a NanoDrop spectrophotometer.

### 2.4. Liquid Chromatography–Tandem Mass Spectrometry (LC-MS/MS)

Peptide samples were analyzed using LC-MS/MS on a Dionex Ultimate 3000 nanoRSLC system coupled to a Thermo Scientific Q Exactive HF-X Orbitrap mass spectrometer (Thermo Fisher Scientific, Waltham, MA, USA). Direct injection was followed by chromatographic separation on a 25 cm C18 analytical column (PepSep, 1.9 µm particles, 75 µm inner diameter) using a one-hour gradient.

Full MS scans were acquired in profile mode, with a scan range of 375–1400 *m*/*z*, resolution of 120,000, an AGC target of 3 × 10^6^, and a maximum injection time of 60 ms. Data-independent acquisition (DIA) was performed using 39 variable windows of 8 Th each, with a resolution of 15,000, AGC target of 3 × 10^5^, maximum injection time of 22 ms, and a normalized collision energy (NCE) of 26. Each sample was run in triplicate to ensure technical reproducibility.

### 2.5. Data Processing Protocol

Orbitrap raw files were analyzed using DIA-NN (Data-Independent Acquisition by Neural Networks) (v19.2), applying a library-free workflow against the UniProt Human Reviewed Proteome (50,516 protein entries; accessed 11 April 2022). The search configuration allowed up to two missed tryptic cleavages and accommodated as many as three variable modifications per peptide. Neural network–based peak detection was employed, and an initial spectral library was generated directly from the DIA data. This library was then used in a secondary round of analysis, implementing a two-pass search strategy.

Within the DIA-NN pipeline, variable modifications considered were methionine oxidation, removal of the N-terminal methionine, and protein N-terminal acetylation, while carbamidomethylation of cysteines was set as a fixed modification. The “match between runs” option was enabled throughout, and precursor identifications were controlled at a 1% false discovery rate. Protein inference was carried out at the gene level, restricted to proteotypic peptides only.

### 2.6. Statistical Analysis

Statistical analyses and visualization were carried out with Perseus (v1.6.15.0). Protein intensities were log2-transformed, organized according to clinical classification, and filtered to retain only those detected in at 50% of the samples. Missing values were imputed from a normal distribution to simulate signals for low-abundance proteins. Group comparisons among ARC–controls, DC–controls, and PVC–controls were conducted using two-sample *t*-tests, and volcano plots were generated with a permutation-based false discovery rate (FDR) threshold of 1% and an S_0_ parameter of 0.1. To assess differences across all cohorts, one-way ANOVA was applied, and results were visualized as heatmaps using the same FDR and S_0_ parameters, with hierarchical clustering based on Euclidean distance.

For gene enrichment analyses, FDR-adjusted *p*-values were calculated in ShinyGO (v0.82) using a permutation-based significance cutoff of 0.01. Functional annotation and pathway mapping were performed through KEGG and Gene Ontology resources, with results illustrated as dot plots and heatmaps [20]. Processed outputs were exported and archived in Excel format for reproducibility.

## 3. Results

### 3.1. Participant Characteristics

In total, 39 individuals were included in the study. In particular, ARC group included 12 patients, DC group included 11 patients, PVC group included 7 patients and control group included 9 patients. The three sample types were collected from each patient. The demographic data of the patients included in the study are presented in Table 1. None of the patients in any group had a history of trauma, ocular inflammation, previous topical or systemic use of steroids or estrogen intake. All patients from the control group had hyperopia prior to lens extraction.

### 3.2. Gene Enrichment Analysis

In total, 1986 proteins were identified in the aqueous humor, 2804 in the anterior capsule, and 3337 in the phaco cassette samples. This high proteome coverage demonstrates the depth and robustness of our DIA-NN–based approach. The extensive protein identifications across different sample types also suggest that each compartment may contribute distinct molecular information relevant to cataract pathophysiology, allowing for subtype-specific insights through gene enrichment and pathway analyses.

#### 3.2.1. Aqueous Humor Samples

**Figure 1:**
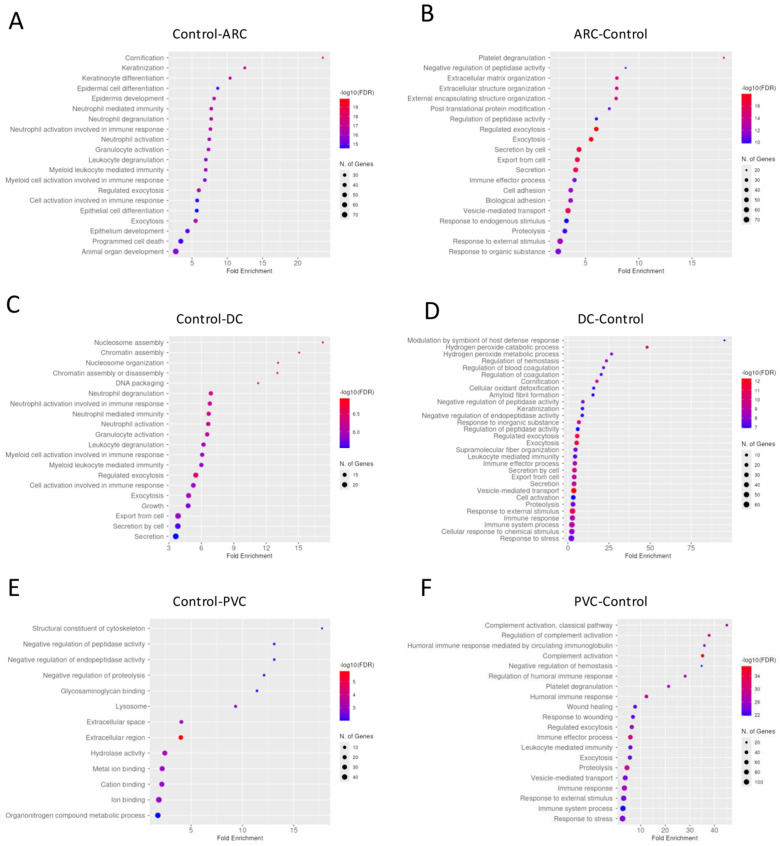
Dotplot of gene enrichment analysis for the aqueous humor proteins that were significantly more abundant in control compared to age-related cataract (ARC) group (**A**), in ARC compared to control group (**B**), in control compared to diabetic cataract (DC) group (**C**), in DC compared to control group (**D**), in control compared to post-vitrectomy cataract (PVC) group (**E**), and in PVC compared to control group (**F**).

**Figure 2:**
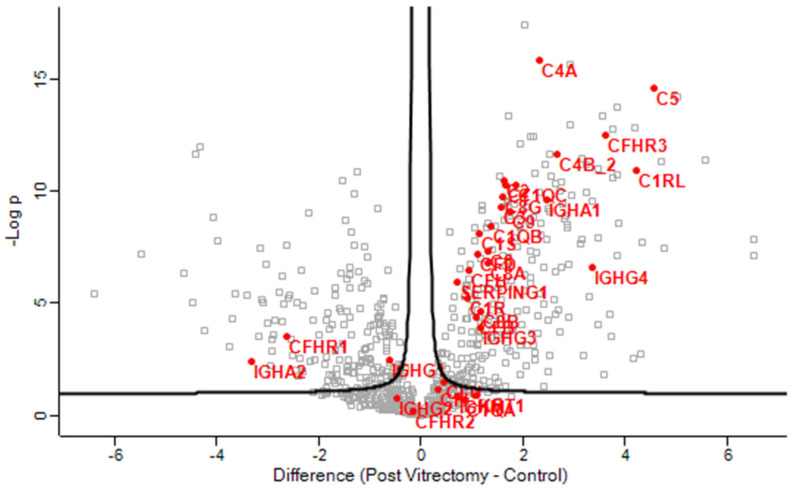
Volcano plot of the aqueous humor sample results, showing the comparison of the proteins involved in the complement activation pathway between post-vitrectomy cataract (PVC) and control group.

Gene enrichment analysis revealed that complement activation pathways were significantly upregulated in the PVC group compared to controls, implicating sustained intraocular inflammation or breakdown of the blood–aqueous barrier post-vitrectomy (Figure 1). Proteins such as C4A and C5—key players in complement activation pathway—were markedly elevated in PVC compared to controls. (Figure 2). Notably, TRAP1 (a mitochondrial chaperone involved in oxidative stress protection), ENO3 (a glycolytic enzyme), and GPM6A (a membrane glycoprotein associated with cellular stress), were significantly downregulated in ARC and DC groups relative to controls.

To improve the dataset consistency, we removed some raw files based on filtering using the “valid values” column, applying a threshold of at least 70% valid values across total measurements. After this filtering, the number of identified proteins per run ranged from 551 (minimum) to 760 (maximum).

To further evaluate data quality and reproducibility, we calculated Pearson correlation coefficients among all runs, which showed values greater than 0.70, indicating good consistency across aqueous humor samples. A heatmap of the Pearson correlation coefficients is included in Supplementary Material S1 to visually demonstrate the data quality.

#### 3.2.2. Anterior Capsule Samples

**Figure 3:**
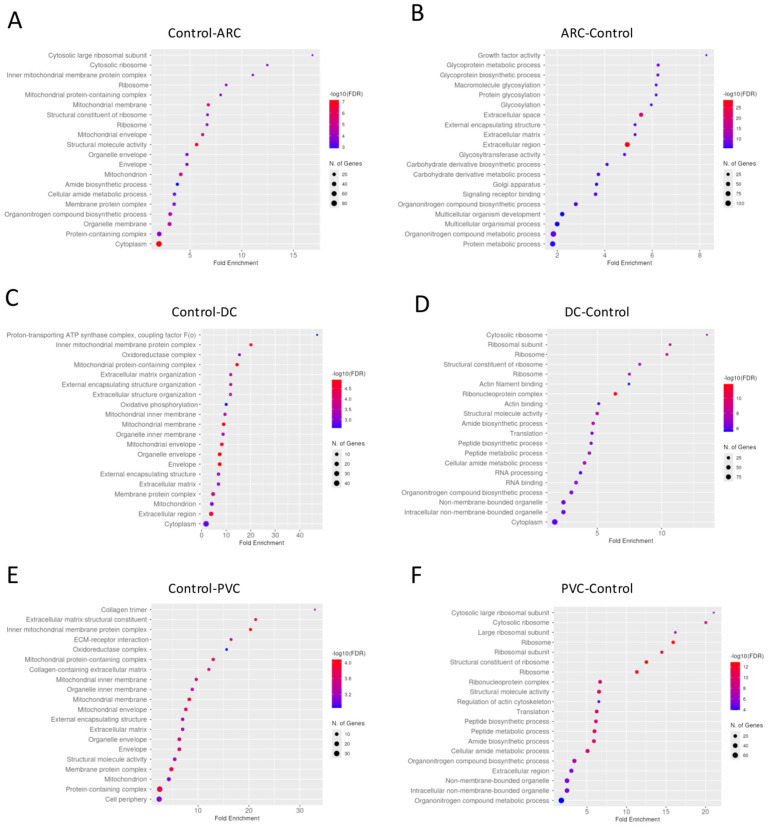
Dotplot of gene enrichment analysis for the anterior capsule samples proteins that were significantly more abundant in control compared to age-related cataract (ARC) group (**A**), in ARC compared to control group (**B**), in control compared to diabetic cataract (DC) group (**C**), in DC compared to control group (**D**), in control compared to post-vitrectomy cataract (PVC) group (**E**), and in PVC compared to control group (**F**).

Gene enrichment analysis of the proteins with significant differential abundance between the groups in anterior capsule samples is shown in Figure 3. To improve the dataset consistency, we removed some raw files based on filtering using the “valid values” column, applying a threshold of at least 50% valid values across total measurements. After this filtering, the number of identified proteins per run ranged from 546 (minimum) to 1031 (maximum).

To further evaluate data quality and reproducibility, we calculated Pearson correlation coefficients among all runs, which showed values greater than 0.50, indicating good consistency across anterior capsule samples. A heatmap of the Pearson correlation coefficients is included in Supplementary Material S1 to visually demonstrate the data quality.

**Figure 4:**
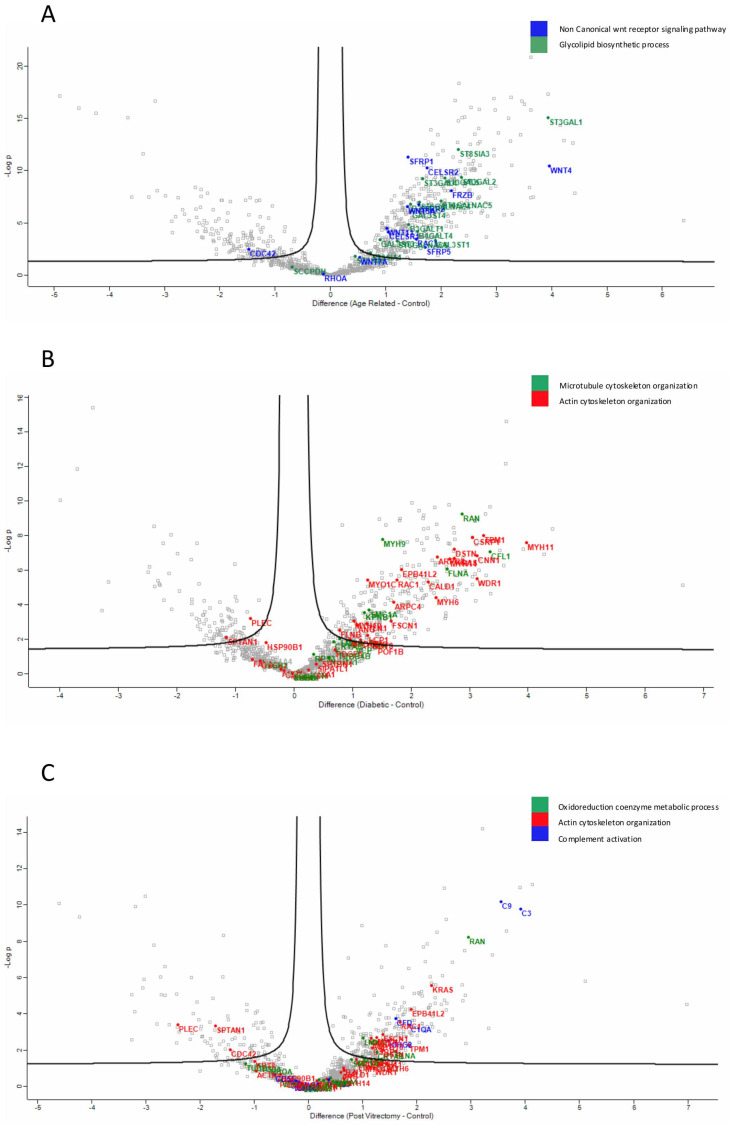
Volcano plot of anterior capsule samples results, showing the comparisons between age-related cataract (ARC)–control (**A**), diabetic cataract (DC)–control (**B**), and post-vitrectomy-cataract (PVC)–control (**C**).

**Figure 5:**
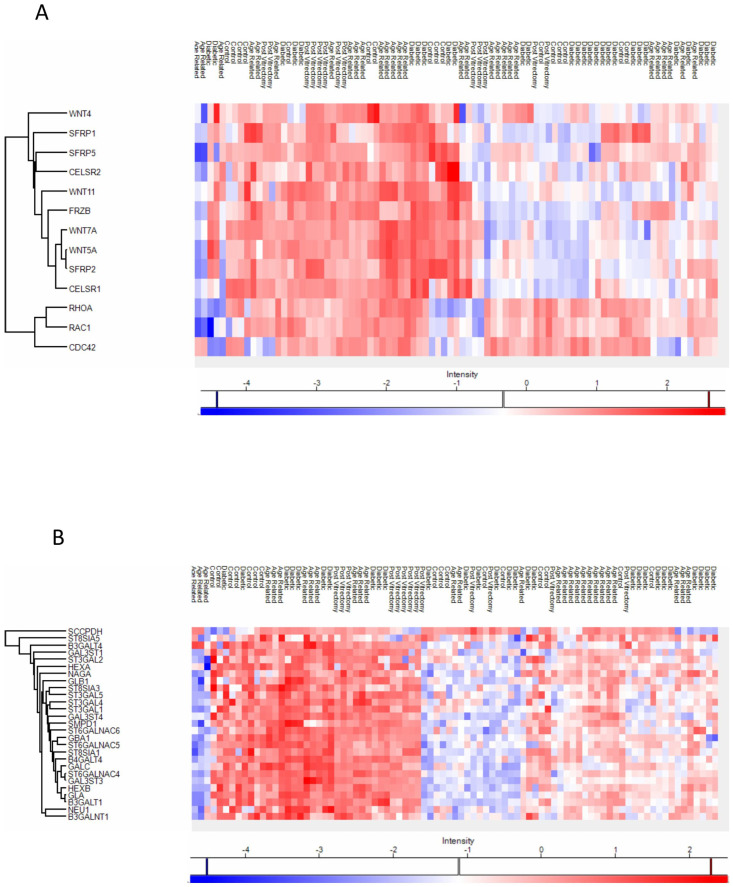
Anterior capsule samples heatmaps of proteins involved in the non-canonical wnt receptor signaling pathway (**A**) and in the glycolipid metabolic process pathway (**B**).

Proteins involved in the non-canonical Wnt receptor signaling pathway, including WNT4 and FRZB, were upregulated in ARC, compared to controls, highlighting aberrant signaling related to epithelial differentiation and fibrosis. (Figure 4A). Proteins involved in glycolipid biosynthetic process, including ST3GAL1 and ST8SIA3, were upregulated in ARC group compared to controls (Figure 4A). In diabetic cataract samples, pathways related to cytoskeletal organization—including actin and microtubule-associated proteins—were prominently upregulated (Figure 4B). Proteins involved in the oxidoreduction coenzyme metabolic process were upregulated in PVC compared to controls (Figure 4C). The most significantly different pathways between the groups in the anterior capsule samples are shown in Figure 4 and Figure 5.

#### 3.2.3. Phaco Cassette Content Samples

**Figure 6:**
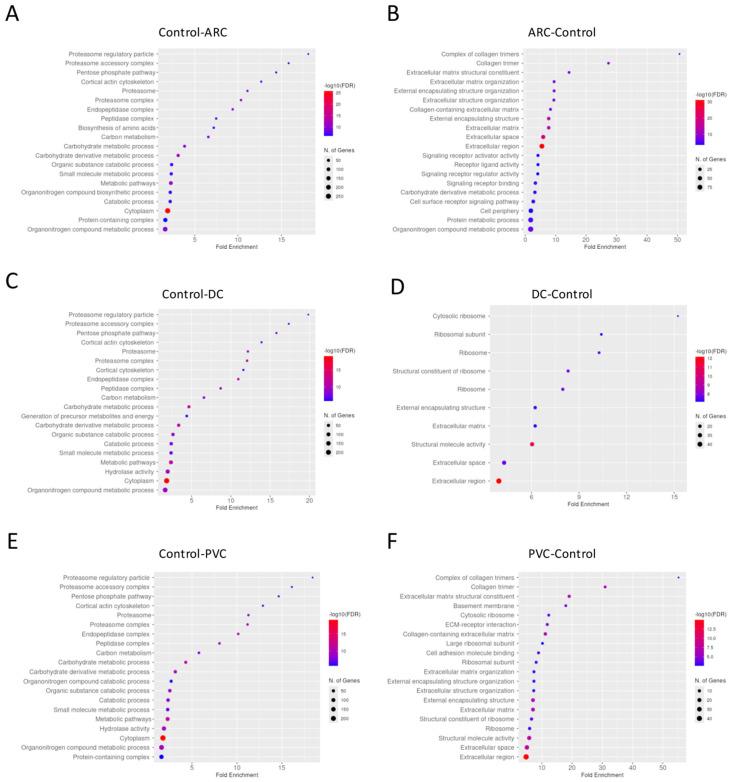
Dotplot of gene enrichment analysis for the phaco cassette content proteins that were significantly more abundant in control compared to age-related cataract (ARC) group (**A**), in ARC compared to control group (**B**), in control compared to diabetic cataract (DC) group (**C**), in DC compared to control group (**D**), in control compared to post-vitrectomy cataract (PVC) group (**E**), and in PVC compared to control group (**F**).

Gene enrichment analysis performed of the proteins with significant differential abundance between the groups in phaco cassette content samples is shown in Figure 6.

**Figure 7:**
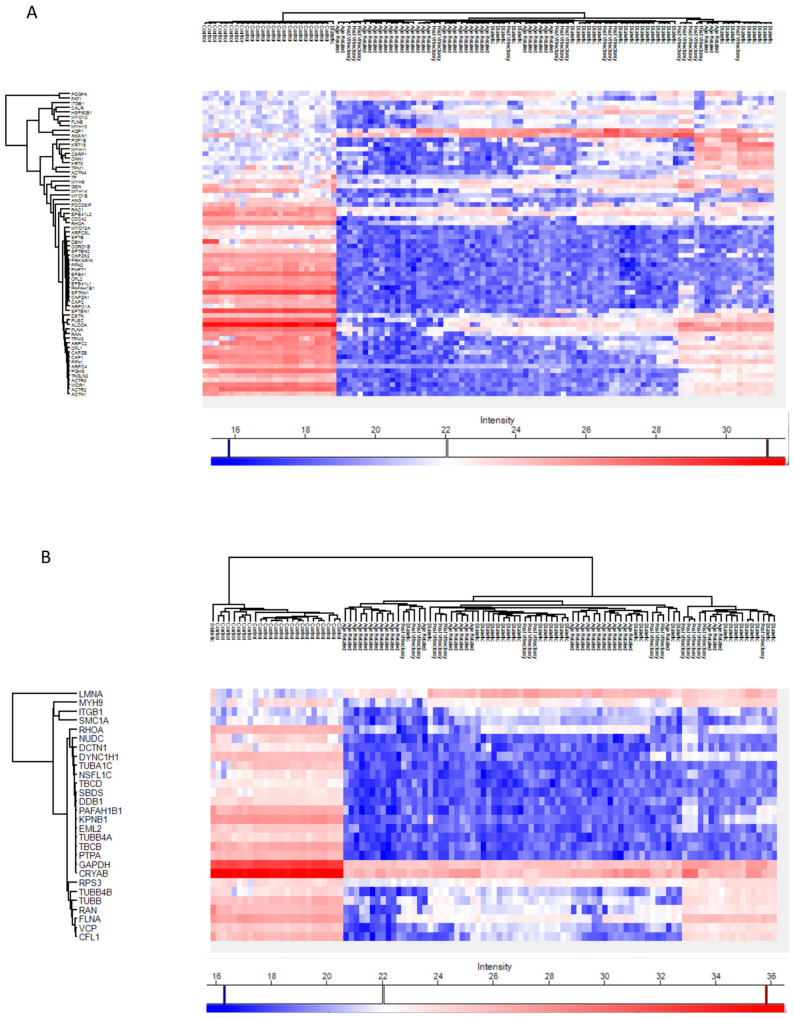
Heatmaps of the Phaco Cassette content sample proteins involved in the actin cytoskeleton organization pathway (**A**) and in the microtubule cytoskeleton organization pathway (**B**).

Across all cataract groups, actin and microtubule cytoskeletal proteins (ACTN4, DCTN1, TUBA1C, TBCB, TUBB4A) were significantly downregulated compared to controls, with the most marked suppression in ARC (Figure 7).

**Figure 8:**
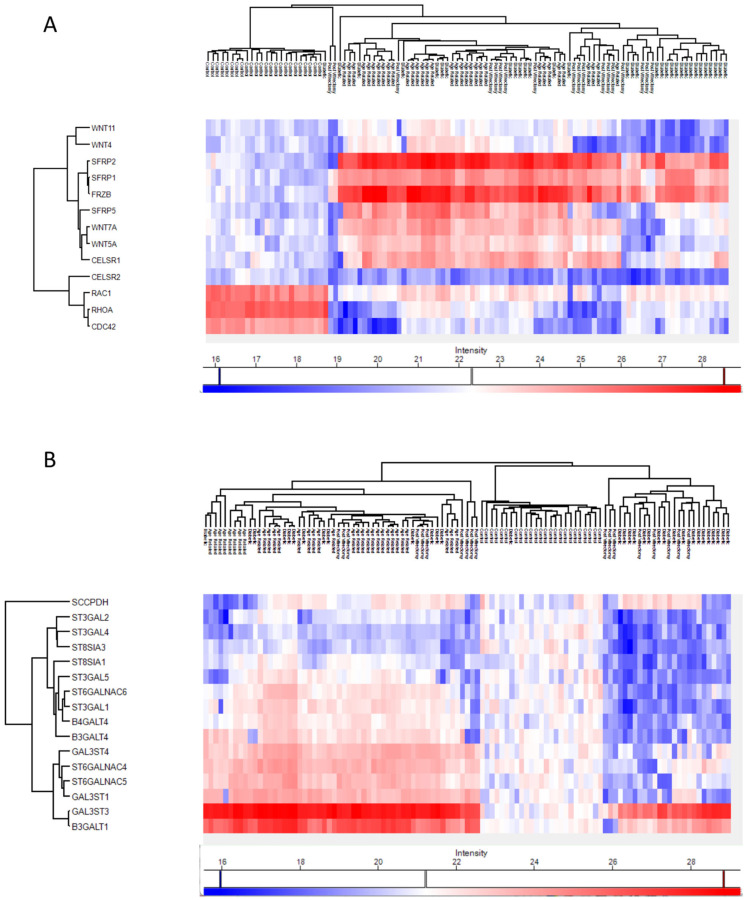
Heatmaps of the Phaco Cassette content samples proteins involved in the non-canonical wnt receptor signaling pathway (**A**) and in the glycolipid biosynthetic process (**B**).

In ARC group, proteins involved in the non-canonical Wnt receptor signaling pathway, including FRZB, SFRP1, SFRP2, SFRP5, WNT5A, and WNT7A, were upregulated compared to DC, PVC, and controls (Figure 7A). Interestingly, while glycolipid biosynthesis pathways were upregulated in ARC (e.g., GAL3ST1, GAL3ST4, GLA), they were markedly downregulated in DC and PVC groups (Figure 8B).

**Figure 9:**
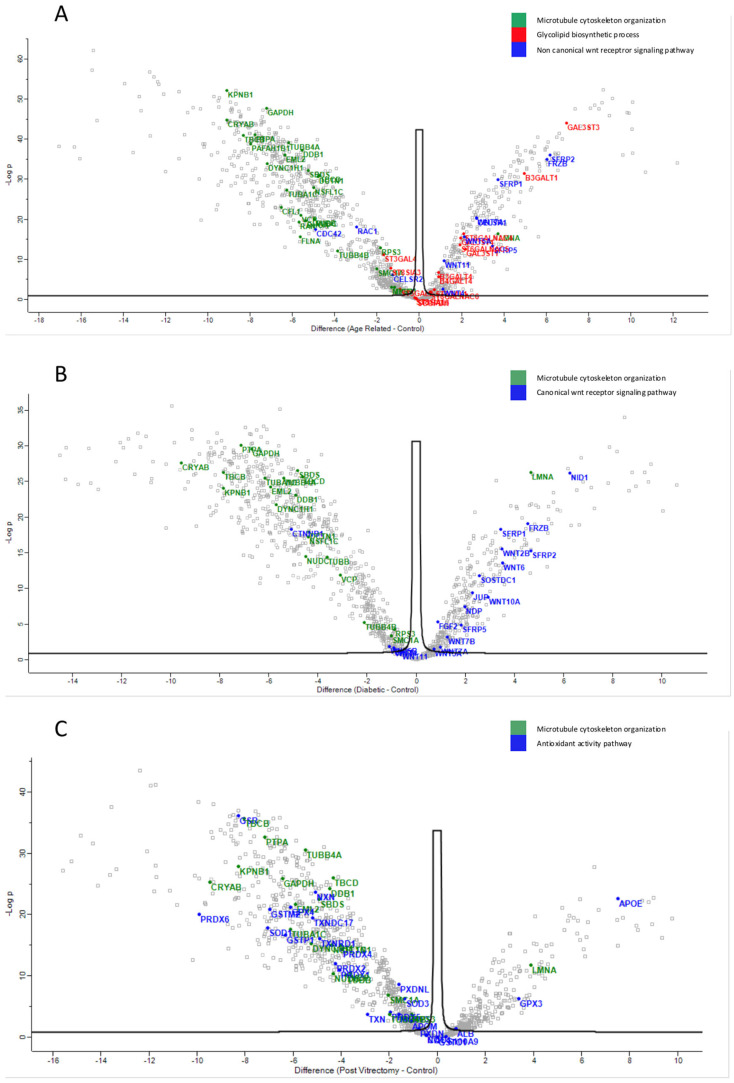
Volcano plot of phaco cassette content samples results, showing the comparisons between age-related cataract (ARC)–control group (**A**), diabetic cataract (DC)–control group (**B**), and post-vitrectomy-cataract (PVC)–control group (**C**).

The most significantly different pathways between the groups in the phaco cassette content samples are shown in Figure 9.

All significant proteins between the four groups in all sample types are summarized in the Supplementary Material S2.

## 4. Discussion

This study provides a comparative proteomic analysis of aqueous humor, anterior lens capsule, and phacoemulsification cassette content across different cataract subtypes and healthy controls. To our knowledge, this is one of the first studies to simultaneously assess these three ocular sample types in age-related cataract (ARC), diabetic cataract (DC), post-vitrectomy cataract (PVC), and healthy eyes, offering novel insights into the molecular pathways underlying distinct cataract phenotypes. In total of 1986, 2804, and 3337 proteins were identified in aqueous humor, anterior capsule, and phaco cassette samples, respectively. This high proteome coverage demonstrates the feasibility and robustness of the DIA-NN-based library-free approach used in this study, which allowed for deep profiling of human aqueous humor and lens proteome. Our findings reveal both shared and distinct molecular alterations across compartments and lens groups, offering insights into cataract subtype-specific pathophysiology and potential mechanisms of lens opacification.

ARC group exhibited upregulation of non-canonical Wnt signaling components (e.g., WNT5A, FRZB, SFRP1/2/5) and proteins involved in glycolipid biosynthesis (ST3GAL1, GAL3ST1). Non-canonical Wnt signaling is critical in maintaining lens epithelial cell polarity and inhibiting epithelial–mesenchymal transition (EMT), a process implicated in lens fiber cell transdifferentiation and opacification [21]. Dysregulation in this pathway may reflect impaired lens epithelial cell polarity, dysregulated differentiation, and compensatory changes to maintain membrane integrity [22,23,24,25,26]. Its upregulation in the ARC group may reflect a compensatory response to early cellular disorganization or adaptive remodeling of lens membranes under oxidative stress, reflecting the altered glycosylation dynamics associated with aging. Similarly, a previous study showed that multiple Wnt ligands and Frizzled receptors were upregulated during TGFβ-induced cataract formation, with consistent findings in both in vitro and in vivo models and that the increased expression of these components may also reflect activation of non-canonical Wnt signaling, particularly the Wnt/Planar Cell Polarity (PCP) pathway, which is crucial for cytoskeletal remodeling [21,26].

Previous studies demonstrated that human cataractous lenses were found to accumulate high levels of Lewis^a^ (Le^a^) glycolipids—molecules typically associated with tumors and early embryonic development. Three specific Le^a^ glycolipids, including sialosylated forms, were identified as major components in cataractous lenses, with their levels increasing with age and cataract progression. Although their exact role was unclear, it was suggested that these glycolipids may influence cell recognition processes involved in cataract formation [27,28]. The increase in glycolipid biosynthesis-related enzymes seen in the ARC group may represent compensatory mechanisms to support membrane integrity or reflect lens-specific glycosylation changes associated with oxidative stress [29].

Intermediate filaments (IFs) are essential components of the cytoskeleton that help connect cells within tissues, including those in the eye lens. A recent review examined the lens-specific IF proteins BFSP1 and BFSP2, highlighting their importance in maintaining lens transparency and optical properties, as well as their involvement in cataract formation and potential roles in lens aging [30]. Evidence generated in studies in both mice and humans suggested a critical role for these proteins and their filamentous polymers in establishing the optical properties of the eye lens and in maintaining its transparency [31,32]. The actin and microtubule cytoskeleton organization proteins were downregulated in all cataract groups compared to controls, but the most marked suppression was seen in the ARC group. Specifically, proteins including ACTN4, DCTN1, MYO18A, TUBA1C, TBCB, and TUBB4A, were downregulated in all cataract groups compared to controls. This may reflect loss or degradation of cytoskeletal integrity in advanced lens opacities [33]. The lens relies on an ordered cytoskeletal architecture to maintain transparency and biomechanical properties [34]. Disruption of these proteins may impair intercellular communication via gap junctions and damage the lens fiber cell architecture, promoting opacification [34]. The convergence of these cytoskeletal alterations across subtypes suggests a common downstream mechanism of lens degeneration. This aligns with earlier research showing that actin disruption hinders lens epithelial elongation and differentiation [35,36]. Our previous findings also suggested that reduced GSN and DAG1 expression may impair the actin cytoskeleton, weakening cell adhesion and lens fiber integrity, and promoting cataract formation [37].

PVC samples exhibited increased abundance of proteins involved in oxidoreduction coenzyme metabolism, consistent with elevated oxidative stress following posterior segment surgery [18,38]. Notably, the PVC aqueous humor was enriched in complement and inflammation-related proteins (C1QC, KNG1, VTN), suggesting persistent low-grade inflammation or compromised blood–aqueous barrier integrity post-vitrectomy. Chronic inflammatory activation may accelerate cataract formation by promoting oxidative injury and extracellular matrix remodeling [11,39,40]. These findings support a distinct immune-mediated mechanism for post-vitrectomy cataracts, differing from the oxidative and metabolic stress pathways predominant in ARC and DC, respectively.

The distinct proteomic signatures identified across ARC, DC, and PVC emphasize that cataractogenesis is not a uniform degenerative process but a condition modulated by subtype-specific drivers—possibly oxidative stress in ARC, cytoskeletal remodeling and metabolic imbalance in DC, and inflammatory activation in PVC. These molecular patterns mirror known clinical and pathological differences among cataract subtypes and could serve as a foundation for subtype-specific therapeutic strategies. Differentially abundant proteins, such as GSN, WNT5A, and FRZB, may serve as candidates for further exploration as diagnostic or prognostic biomarkers given their consistent subtype association and mechanistic relevance to lens homeostasis.

A key consideration in interpreting our findings is the role of proteoforms, which arise from post-translational modifications, alternative splicing, or sequence variants. Lens proteins are particularly susceptible to oxidative and glycation modifications, which can affect stability, aggregation, and light-scattering properties even without major abundance changes. Validation of candidate proteins such as WNT5A, ACTN4, or FRZB in lens models may clarify their mechanistic roles in cataractogenesis and guide potential interventions. Future studies employing top-down or hybrid proteomic approaches to resolve proteoforms will be critical for fully capturing cataract subtype complexity and identifying clinically relevant biomarkers or therapeutic targets.

Recent studies highlight important advances in the field of cataract research. Hubbard & Shoff et al. identified over 200 new isomerization sites, mainly in noncrystallin proteins, including modifications at aspartic acid, serine, glutamic acid, and notably histidine, which was linked to potential metal binding [41]. The study overall showed that isomerization increases with age and occurs more readily in flexible, unstructured regions, and that combining data from multiple isomerized peptides can accurately predict tissue age, suggesting new forensic and biological applications for isomerization profiling [41]. Zelle et al. applied diaPASEF mass spectrometry to zebrafish lenses, revealing region-specific protein expression patterns and validating a powerful approach for deep proteomic analysis of lens aging [42]. Wang et al. showed that significant modifications to human lens γ-crystallins, including truncation and deamidation, are linked to protein aggregation and increased membrane association in cataractous lenses [43]. These changes appear to play a direct role in cataract formation, as they occur only at minimal levels in age-matched normal lenses [43]. Cantrell et al. demonstrated that age-dependent shifts in lens metabolism and decreases in calcium-dependent cell–cell junctions, such as connexins, which likely impair fiber-cell permeability and microcirculation, contributing to nuclear cataract formation [44]. Schey et al. visualized crystallin-derived peptide distributions in normal versus cataractous lenses, detecting specific γS-crystallin truncations associated with cataract [45].

Collectively, these studies complement our findings by highlighting structural, metabolic, and post-translational changes in lens proteins as key contributors to cataractogenesis, guiding future research in the field.

## 5. Conclusions

Our study provides a comprehensive proteomic characterization of ARC, DC, and PVC, revealing both shared and divergent molecular mechanisms underlying cataract formation. While oxidative stress, inflammation, and cytoskeletal remodeling appear to be common themes, specific pathways—such as complement activation in PVC or glycolipid metabolism in ARC—appear to be subtype-specific and may represent potential therapeutic targets. The identification of differentially abundant proteins such as GSN, WNT5A, and FRZB across sample types further supports the involvement of diverse biological processes in cataractogenesis and provides a foundation for future target validation. The comprehensive proteomic profiling presented here sets the stage for future studies integrating transcriptomic, metabolomic, and clinical data to achieve a more holistic understanding of cataract pathophysiology.

## 6. Limitations

This study should be interpreted in light of its limitations. All cataract subtypes were grouped together to enable a broad investigation into the general pathways involved in cataractogenesis. However, analyzing individual cataract subtypes separately could yield valuable insights into the distinct molecular mechanisms underlying each form. Additionally, the phaco cassette samples were collected post-phacoemulsification, a procedure that potentially induces oxidative stress through free radical generation. Nevertheless, the analysis of such samples may offer novel opportunities for methodological advancements in cataract research. Moreover, the control group was not strictly age-matched to the ARC cohort. Consequently, some of the proteomic differences observed in the ARC group may reflect normal aging processes rather than cataract-specific alterations. However, because clear human lenses from individuals aged 70–80 years are exceedingly rare, we recruited controls at a somewhat younger age (<60 years) to ensure that the lenses were free of cataractous changes. This also allowed the control group to be more appropriately age-matched to the DC and PVC cohorts. Finally, further studies are warranted to validate the identified pathways and key proteins using orthogonal approaches, such as targeted proteomic analyses or independent patient cohorts, to confirm their biological significance and strengthen the robustness of these findings.

## Figures and Tables

**Table 1:** Clinical and demographic characteristics of the participants.

	ARC Group	DC Group	PVC Group	Control Group
Subjects	12	11	7	9
Mean age (years, mean ± SD)	79.6 ± 4.2	61.7 ± 4.3	60 ± 10.2	56 ± 3.3
Sex (male/female)	5:7	7:4	2:5	4:5
OD:OS	6:6	7:4	1:6	7:2
Dominant type of cataract (NS:CS:PSC)	8:2:2	5:1:5	3:0:4	N/A
Mean height (cm, mean ± SD)	164.08 ± 9.98	171.45 ± 7.13	168.88 ± 7.08	173.56 ± 7.94
Mean weight (kg, mean ± SD)	70.58 ± 13.75	94.55 ± 6.82	77.88 ± 13.35	74.11 ± 13.63
Mean AL (mm, mean ± SD)	23.69 ± 0.97	23.10 ± 0.80	24.52 ± 1.49	21.73 ± 1.22
Mean K1 (D, mean ± SD)	42.89 ± 1.44	43.02 ± 0.64	41.51 ± 1.51	42.06 ± 0.55
Mean K2 (D, mean ± SD)	43.58 ± 1.55	44.17 ± 0.79	42.65 ± 1.61	42.87 ± 0.87
Mean sun exposure (hours, mean ± SD)	2.2 ± 1.5	3.5 ± 2.5	4.3 ± 2.4	1.0 ± 1.32
Use of sunglasses (Yes/No)	6:6	7:4	3:4	7:2
Iris color (brown/hazel/blue)	9:3:0	8:2:1	5:1:1	9:0:0
Smoking (Yes/No)	3:9	4:7	0:7	7:2
Alcohol consumption (Yes/No)	2:10	1:10	0:7	8:1
Hypertension (Yes/No)	7:5	2:9	4:3	8:1
Glaucoma (Yes/No)	1:11	0:11	0:7	0:9
Aspirin intake (Yes/No)	1:11	1:10	0:7	3:6
AMD (Yes/No)	4:8	0:11	0:7	0:9
Thyroid disease (Yes/No)	3:9	2:9	0:7	0:9
Diet supplementary intake (Yes/No)	5:7	3:8	1:6	3:6

ARC = Age-related cataract; DC = Diabetic cataract; PVC = Post-vitrectomy cataract; SD = Standard Deviation; OD = Right eye; OS = Left eye; NS = Nuclear sclerotic cataract; CS = corticoid spoking cataract; PSC = Posterior subcapsular cataract; AL = Axial length; K1 = flat meridian of the anterior corneal surface; K2 = steep meridian of the anterior corneal surface; D = Diopters; AMD = Age-related macular degeneration; N/A = Not applicable.

## Data Availability

The mass spectrometry proteomics data have been deposited to the ProteomeXchange Consortium via the PRIDE partner repository with the dataset identifier PXD045547, PXD045554, PXD045557, PXD069667 [46].

## Supplementary Materials

The following supporting information can be downloaded at: https://www.mdpi.com/article/10.3390/proteomes13040062/s1, Supplementary Material S1: *T*-test statistically significant proteins in all three sample types. Supplementary Material S2: Figure S1. Heatmap of Pearson correlation coefficients among all runs, which showed values greater than 0.70, indicating good consistency across all aqueous humor samples. Figure S2. Heatmap of Pearson correlation coefficients among all runs, which showed values greater than 0.50, indicating good consistency across all anterior capsule samples.
