# Supplementary material for: Comparative Proteomic Analysis of Aqueous Humor, Anterior Lens Capsules, and Crystalline Lenses in Different Human Cataract Subtypes Versus Healthy Controls
Source: Proteomes. 2025 Nov 21;13(4):62. doi: 10.3390/proteomes13040062 (PMC12641653; doi:10.3390/proteomes13040062)
Supplement: Supplementary file 1 [file proteomes-13-00062-s001.zip › Supplementary materials S2.pdf]

Supplementary material S1

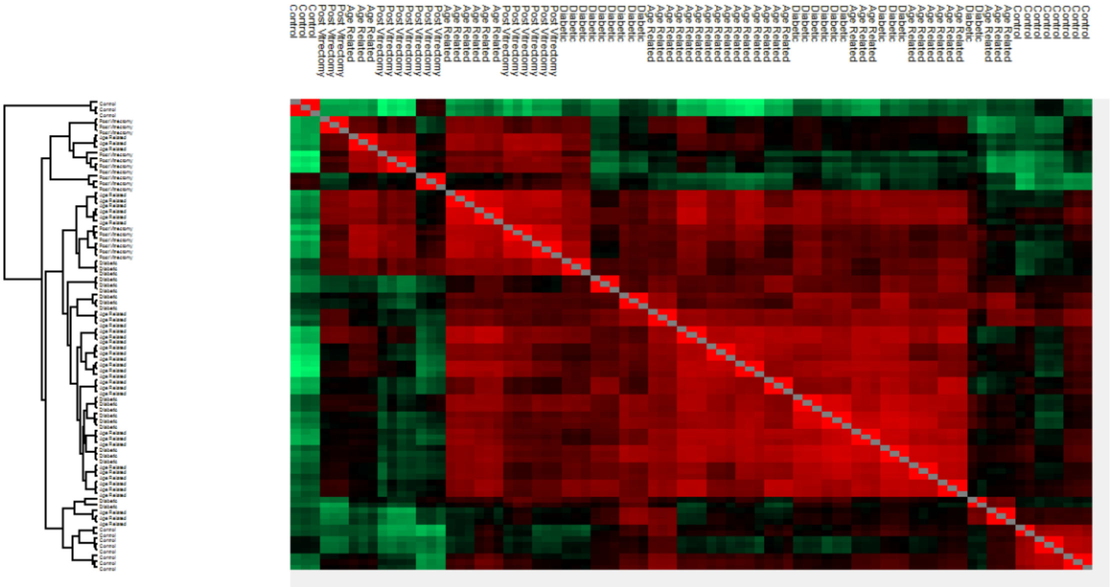

Figure S1. Heatmap of Pearson correlation coefficients among all runs, which showed values greater than 0.70, indicating good consistency across all aqueous humour samples.

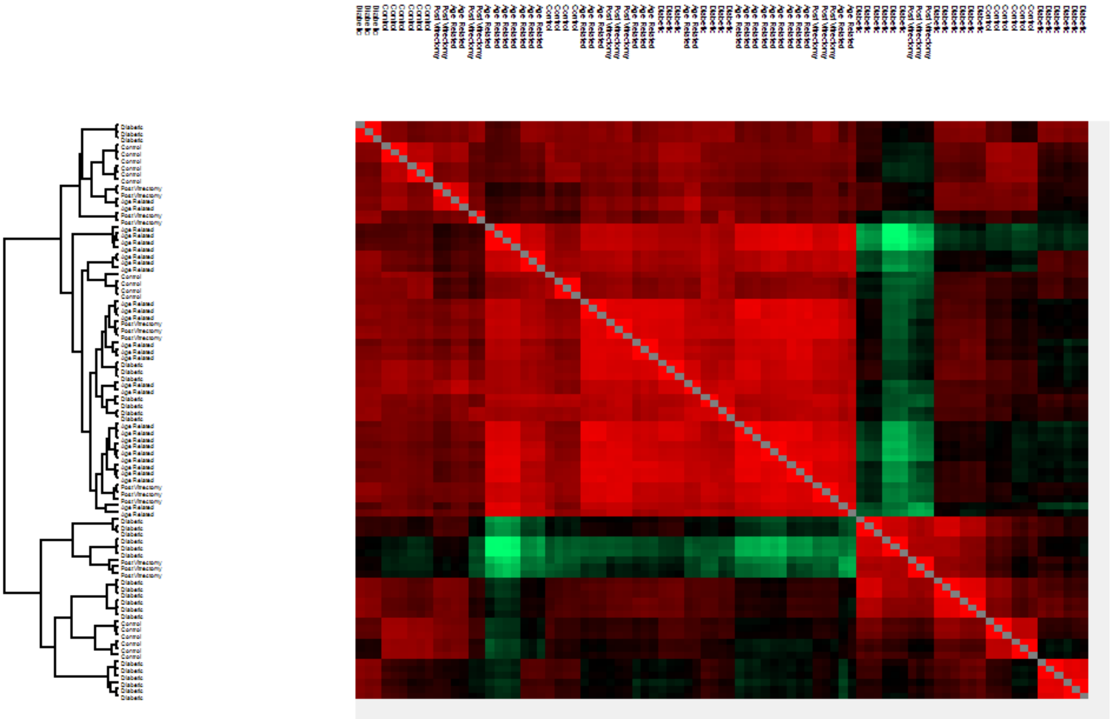

Figure S2. Heatmap of Pearson correlation coefficients among all runs, which showed values greater than 0.70, indicating good consistency across all anterior capsule samples.
